# TUJI1 Dataset: Multi-device dataset for indoor localization with high measurement density

**DOI:** 10.1016/j.dib.2024.110356

**Published:** 2024-03-22

**Authors:** Lucie Klus, Roman Klus, Elena Simona Lohan, Jari Nurmi, Carlos Granell, Mikko Valkama, Jukka Talvitie, Sven Casteleyn, Joaquín Torres-Sospedra

**Affiliations:** a Electrical Engineering Unit, Tampere University, 33720 Tampere, Finland; b Institute of New Imaging Technologies, Universitat Jaume I, 12071 Castellόn de la Plana, Spain; c Departament d'Informàtica, Universitat de València, 46100 Burjassot, València, Spain

**Keywords:** Device heterogeneity, Fingerprinting, Indoor positioning, Localization, RSS, RSSI, Wi-Fi

## Abstract

Positioning in indoor scenarios using signals of opportunity is an effective solution enabling accurate and reliable performance in Global Navigation Satellite System (GNSS)-obscured scenarios. Despite the availability of numerous fingerprinting datasets utilizing various wireless signals, the challenge of device heterogeneity and sample density remains an unanswered issue. To address this gap, this work introduces TUJI1, an anonymized IEEE 802.11 Wireless LAN (Wi-Fi) fingerprinting dataset collected using 5 different commercial devices in a fine-grained grid. The dataset contains the matched fingerprints of Received Signal Strength Indicator (RSSI) measurements with the corresponding coordinates, split into training and testing subsets for effortless and fair reproducibility.

Specifications Table

**Table:** 

Subject	Geographical Information System, Data Mining and Statistical Analysis, Signal Processing
Specific subject area	Fine-grained Wi-Fi fingerprinting for indoor positioning using heterogeneous devices
Type of data	Table
Data collection	The fingerprints were acquired by performing a site survey in the ESPAITEC 2 building at Universitat Jaume I (UJI), Castellón de la Plana, Spain. The data was gathered using 5 commercially available devices, namely mobile devices Samsung Galaxy S20, Samsung Galaxy S7, Xiaomi POCO X3, Samsung Galaxy A12, and a tablet Samsung Galaxy Tab S7+ with the GetSensorData application [1], version 2.1. The local coordinates were acquired using a laser measure BOSCH Professional GLM 120 C. The survey was performed by iteratively measuring the Wi-Fi Received Signal Strength Indicator (RSSI) (standardized received signal strength), among other variables, at the pre-defined locations across multiple offices of different sizes with each considered device during low network traffic hours. The continuous network measurements were taken at each location for a minimum of 10 s and later matched to the coordinates based on the temporal proximity, where valid measurements are taken 3 or less seconds before the marked position.
Data source location	*Institution:* Universitat Jaume I, ESPAITEC 2 building *City/Town/Region:* Castellón de la Plana *Country:* Spain *Latitude and longitude for collected samples/data:* 39.993934360435766, −0.07402203156600276
Data accessibility	Repository name: Zenodo Data identification number: 10.5281/zenodo.7641701 Direct URL to data: https://zenodo.org/record/7641701[2]

## 1 Value of the Data

• Recent progress in deep learning and artificial intelligence has opened new opportunities in indoor positioning research. However, the lack of high-quality, representative datasets presents one of the main challenges within this field. Uniqueness of scenarios, high variety of utilized devices, and numerous site-surveying strategies further impact the requirements on the available data, and search for common approaches. Compared to the other indoor positioning, fingerprinting datasets utilized in e.g. [3], [4], [5], [6], the TUJI1 dataset is unique in two main aspects:
  • It considers very fine measurement granularity, with data points spaced less than 45 cm apart, enabling studying the impact of data density on the positioning performance.
  • It investigates effects of device heterogeneity, while utilizing 5 distinct devices for data acquisition. The device labels are available for all samples, allowing the assessment of performance variation across devices and leading to the development of methods balancing such variations.
  Additionally, the raw data was pre-formatted for effortless implementation as an indoor positioning dataset. As assessing the performance of a tested model on multiple datasets demonstrates the model's generalization properties – particularly in indoor positioning scenarios, where is deployment is unique - this manuscript also provides a benchmark performance. TUJI1 can also be generalized as a benchmark for evaluating the performance of general regression models within the machine learning community, with pre-allocated features and labels, as well as train/test split. Moreover, the dataset can be utilized to model and study Wi-Fi signal propagation in indoor environment, as it includes coverage maps of over 300 distinct access points (APs). The availability of raw data enables further applications in e.g. dimensionality reduction or unsupervised learning studies.

## 2 Background

As hundreds of different devices operate within modern radio networks, the suitability of crowdsourcing and crowdsensing solutions and the challenges these techniques introduce are of utmost relevance. In such crowdsourcing scenarios, the data is collected by network users, while only their radio measurements and locations are recorded. The physical locations of each Access Point (AP) or the general deployment geometry are unknown. A fingerprinting dataset comprised of such measurements is suitable for training the data-driven models, such as neural networks, support vector machines, decision trees, etc. The TUJI1 dataset was furthermore specifically developed to study the effects of the measurement granularity and device heterogeneity, two of highly important aspects when using wireless networks in the scope of indoor positioning. In this work, data granularity refers to the density of the data points within the physical area of interest. The term device heterogeneity describes the variations in individual devices’ performance, characteristics, etc. Moreover, the indoor positioning community calls for additional data on which the models and methods can be evaluated, as a data collection campaign is time-intensive, and each deployment differs in terms of physical and network characteristics, mobility, number of measurements, as well as utilized devices and technologies to obtain the data. The raw data was formatted to enable effortless applicability by the scientific community, and is provided in universal .csv format, as well as a MATLAB structure.

## 3 Data Description

The provided data files are presented below:

• Formatted “TUJI1” dataset: “DATASET\TUJI1.mat” corresponds to the MATLAB structure “database”, containing four matrixes. The structure is consistent with the positioning datasets published and evaluated in [3,7]. The “database.trainingMacs” and “database.testMacs” correspond to the matrixes of training and testing features (RSSIs), respectively. Columns specify the access point indexes, while rows define the sample indexes. The “database.trainingLabels” and “database.testLabels” matrixes define the training and testing labels, respectively. There, the first three columns denote the coordinates in “x”, “y”, and “z” axes, fourth and fifth columns denote floor and building index, while the sixth column denotes the device label (see Table 2).
• Training features: “DATASET\RSS_training.csv” is a text file and corresponds to the “database.trainingMacs” matrix in accessible .csv format. There are as many rows as fingerprints in the training set, while the number of columns corresponds to the number of Wi-Fi APs detected within the dataset (AP1 to AP310).
• Testing features: “DATASET\RSS_testing.csv” is a text file and corresponds to the “database.testMacs” matrix in accessible .csv format. There are as many rows as fingerprints in the testing set, while the number of columns corresponds to the number of Wi-Fi APs detected within the dataset (AP1 to AP310).
• Training labels: “DATASET\Coordinates_training.csv” is a text file which corresponds to the “database.trainingLabels” matrix in accessible .csv format. There are as many rows as fingerprints in the training set, while the columns represent the coordinates in “x”, “y”, and “z” axis, followed by the floor and building index. The sixth column denotes the device label (see Table 2).
• Testing labels: “DATASET\Coordinates_testing.csv” is a text file which corresponds to the “database.testLabels” matrix in accessible .csv format. There are as many rows as fingerprints in the testing set, while the columns represent the coordinates in “x”, “y”, and “z” axis, followed by the floor and building index. The sixth column denotes the device label (see Table 2).
• Device labels: “DATASET\Device_labels.csv” is a text file which refers to the table “device_labels” linking the device indexes with the devices themselves according to Table 2.
• Raw data files: “RAW\AAA\AAA_LogFiles_GetSensorData\logfile_2022_MM_DD_HH_mm_SS.txt” correspond to the raw measurements acquired from the GetSensorData [1] application and contain logs of the measured entities, along with a header specifying the application version and the device. The “AAA” corresponds to the device name, “MM” specifies the month, “DD” the day, “HH” the hour, “mm” the minute, and “SS” the second of the beginning of the measurement. The data are stored in logfiles in plain text. Each logfile corresponds to a single data collection session and has as many rows/lines as sensor readings/measurements. The format for each line depends on the sensor that provided that reading, and the sensor type is identified in the first four characters. The format to retrieve data from each sensor type is included in the header lines in every logfile. The Wi-Fi and Bluetooth Low Energy (BLE) measurements within the raw data were completely anonymized (Media Access Control address (MAC) and Service Set Identifier (SSID) for Wi-Fi and MAC, Universally Unique Identifier (UUID), Major ID and Minor ID for BLE).
• The table mapping the coordinates to the measurements: “map2coordinates.xlsx” contains the tables mapping the position marks from the log files to the physical coordinates, specified in “map2coord test” and “map2coord train” sheets.
• Pre-formatted data: “FORMATTED\Formatted_data_AAA.mat” correspond to the structures with pre-formatted samples from the log files of the “AAA” device, containing the detected access points’ MAC address and measured RSSIs, along with the physical coordinates. The files contain separate structures for training and testing data.
• The script to format raw data: “FormatRawData.m” defines the parameters for the data formatting and transforms the raw logs into pre-formatted data.
• Log-reading function: “readrawfile.m” provides a function for reading and extracting relevant information from the log files. The function was based on the scripts available at “https://gitlab.com/getsensordatasuite”.
• Linking function: “makedataset.m” transforms the extracted measurements and the corresponding labels into an organized structure.
• The script to generate the “TUJI1” dataset: “CreateDataset.m” provides the means to transform the pre-formatted data into a standardized dataset.

## 4 Experimental Design, Materials and Methods

The description of the deployment, utilized devices and application, survey methodology, and benchmark results are presented in the paragraphs below.

### 4.1 Scenario of interest, utilized devices and application

**Fig. 1:**
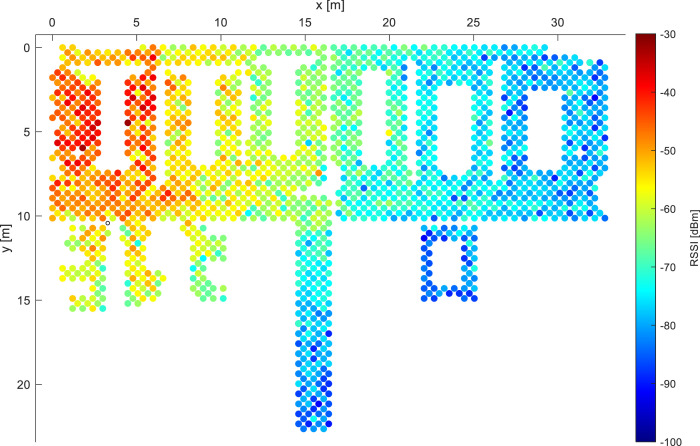
Example 1 of RSSI distribution for a single AP while utilizing data from all devices.

**Fig. 2:**
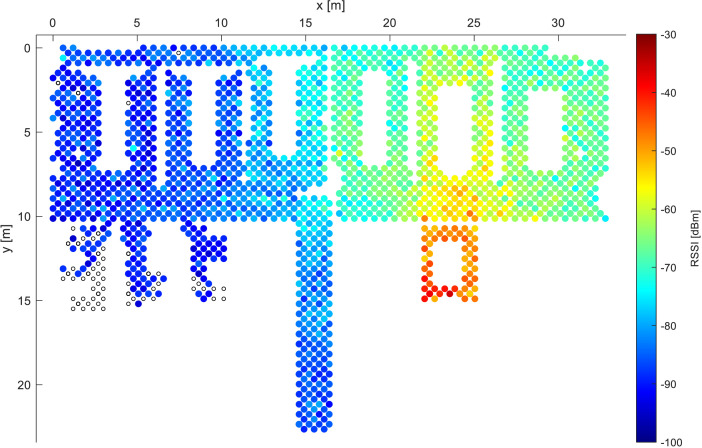
Example 2 of RSSI distribution for a single AP while utilizing data from all devices.

**Fig. 3:**
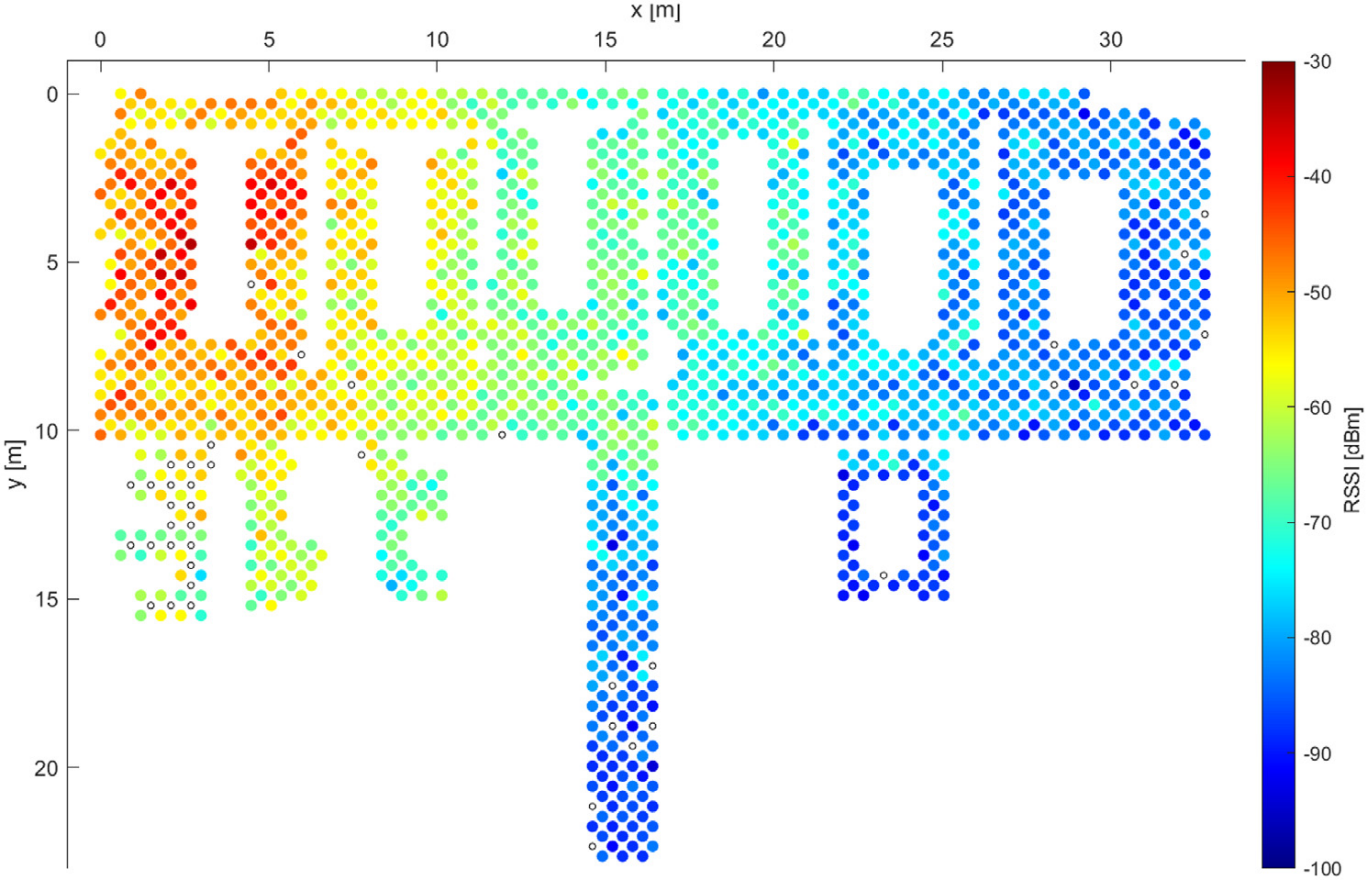
Example 2 of RSSI distribution for a single AP while utilizing data from Galaxy S20 device only.

**Fig. 4:**
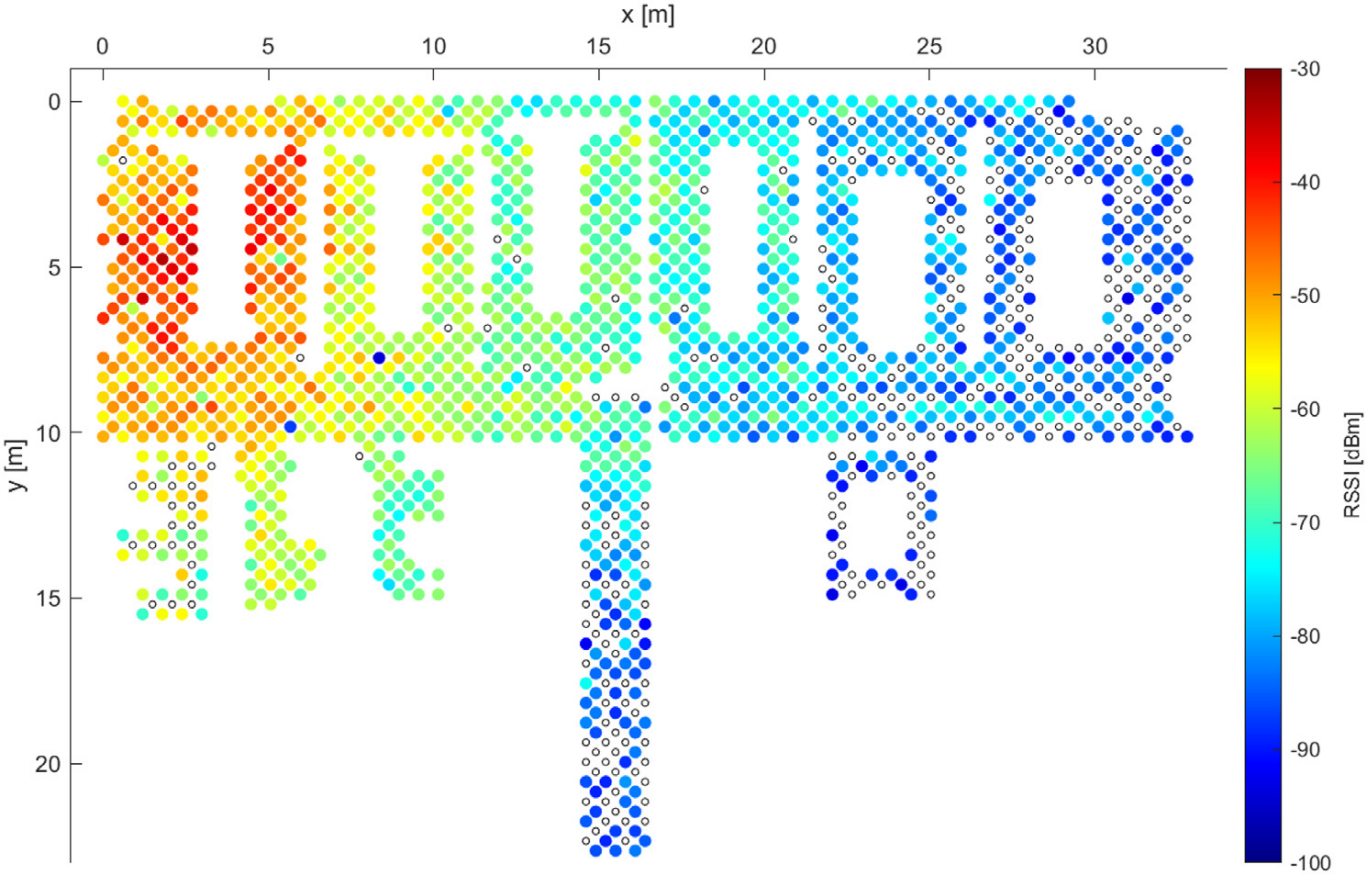
Example 2 of RSSI distribution for a single AP while utilizing data from Galaxy S7 device only.

The site of interest covers multiple offices of different sizes and a corridor on the fifth floor of Espaitec2 building of UJI, Castellón de la Plana, Spain. This 5-floor building is a regular office building with plasterboard walls. The site-survey was realized on the top floor, under the roof with solar panels and outdoor air conditioning units to warm/cool the entire building. The collection environment considers two separate laboratory areas at the north of the building, located side by side and separated by a thin plasterboard wall, with 3 small offices each (6 in total, 2 of which were not accessible), covering an total area of 32.8 m by 22.6 m (floor map in Fig. 1). In each laboratory area, 3 large office tables are present, separated by a series of wooden office cupboards (i.e., respectively the larger and thinner white spaces in Fig. 1, Fig. 2, Fig. 3, Fig. 4). Heading south from the entrance to the two laboratories is a corridor leading to two elevators in the middle and two similar laboratories at the south side of the building. The wireless environment can be characterized as varying line-of-sight (LoS) and non-line-of-sight (NLoS) coverage from a multitude of access points available in the area, with parts of deployment being well covered, while others having poor radio link conditions. In wireless networks, the LoS and NLoS conditions determine the existence (or its lack) of the direct signal path from the transmitter to the receiver, which greatly determines the signal behaviour. The measured entity considered in the dataset is the Wi-Fi Received Signal Strength (RSS), determining how much power was received by the device. More specifically, the measurement of RSS is within the current Wi-Fi standards and Android systems obtained as RSSI, an indicator of power received within the signal. In the measurement process (and withing the raw data) more entities were collected, including BLE RSS, accelerometer data, or the GNSS signals. During the site survey, almost 9 000 individual valid fingerprint samples were collected with the mobile application GetSensorData [1] version 2.1. and later labelled with the corresponding coordinates.

The general information listing the number of samples, number of APs (unique MAC addresses), etc. can be found in Table 1.

**Table 1:** General information.

Number of fingerprints	8899
Number of Wi-Fi RSSI measurements	196,629
Number of samples in training set	6752
Number of samples in testing set	2147
Number of detected access points	310
Number of measurements per location	5
Measured entity	Wi-Fi RSSI

The specific examples of the radio coverage of the deployment are depicted in Fig. 1 and Fig. 2, characterizing the RSSIs from two selected access points. The black circles in the figures denote locations with no valid measurements from the given AP. The outline of the deployment visualized in the figures depicts two large open office rooms, four smaller offices, and a hallway connected to them. The deployment considered in [8] refers to the open office room in the left side of the figure, while we consider finer grid. The figures demonstrate the applicability of the fingerprinting method which can be utilized onto the data, as each access point creates a unique map of the environment, thus in case the device detects the RSSI of −70 dBm from both APs (light blue color), the model most likely determines its location in the central areas of the deployment. Both figures also show significant variations of the measured signal strength, abruptly changing colors in seemingly open areas.

To investigate the effects of signal variations further, Fig. 3 and Fig. 4 depict the signal coverage, as detected by the Galaxy S20 and Galaxy S7 devices, respectively. While in the areas with relatively strong detected RSSI both devices exhibit an almost-perfect coverage (red and yellow colors in the figures), the Galaxy S7 device clearly struggles to detect the signals from the AP when the signal strength is poorer, as is the case in the right side of Fig. 4. This illustrates only one, yet important aspect of the device heterogeneity. Further studies are required for reliable and robust quantization of such effects, and TUJI1 provides solid data to support such research.

The 5 devices used for the site survey include 4 smartphones with varying performance and supported standards, and a tablet. The individual devices are summarized in Table 2, listing the label used in the dataset corresponding to each device, device type, number of locations with valid samples, and the operating system (OS) of the device. Additionally, the number of valid RSSI measurements per sample is included, as well as their mean and standard deviation (std). The table clearly shows the discrepancy of measurements across devices, highlighting more than 20 dBm mean measured RSSI more using Galaxy S20 than Galaxy A12, with significantly higher number of valid measurements.

**Table 2:** List of utilized devices.

Label	Model	Type	OS	Valid RSSIs	Valid samples	RSSIs per sample [mean (std)]
1	Galaxy S20	Smartphone	Android 11	61,471	1828	33.6 (6.2)
2	Galaxy S7	Smartphone	Android 8	40,178	1756	23.3 (5.2)
3	POCO X3	Smartphone	Android 11	40,242	1747	23.6 (5.6)
4	Galaxy Tab S7	Tablet	Android 11	36,595	1835	21.5 (6.2)
5	Galaxy A12	Smartphone	Android 11	18,143	1733	9.9 (3.4)

**Fig. 5:**
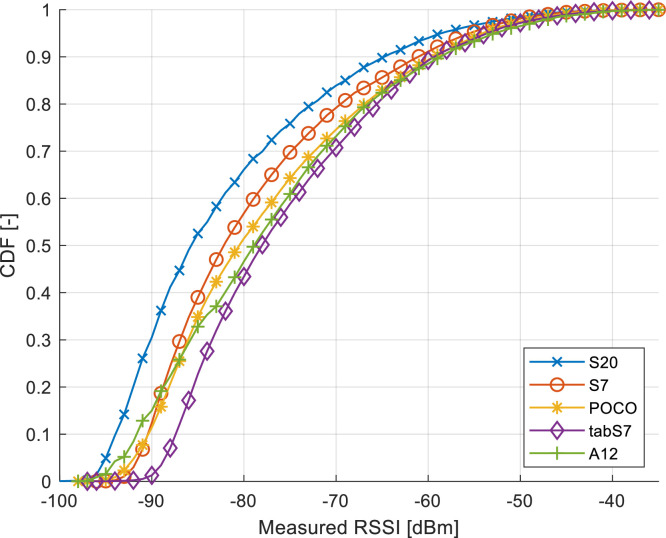
CDF of the RSSI measurements per utilized device.

**Fig. 6:**
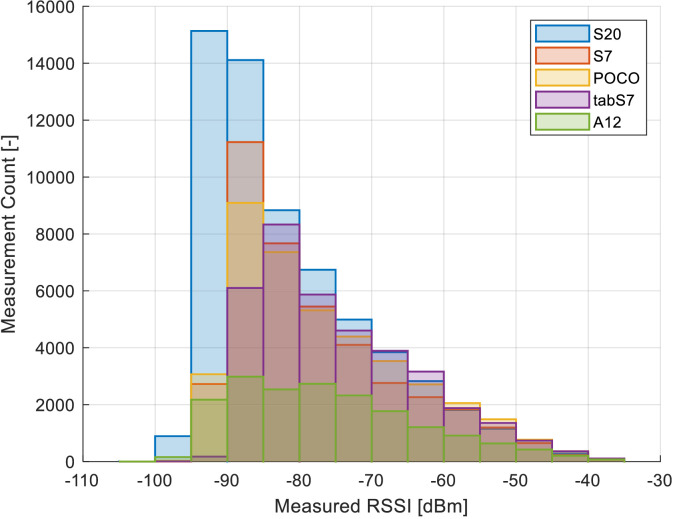
Histogram of the RSSI measurements per utilized device.

The impact of the heterogeneity of the devices is further shown in Fig. 5, which visualizes the cumulative distribution function (CDF) of the measured RSSIs per device. Here, the Galaxy S20 was able to acquire a larger amount of low-quality RSSI measurements than the other devices. Additionally, the histograms of the RSSIs are presented in Fig. 6, showing the discrepancy across devices in the environment across all obtained samples. The figures show that the off-the-shelf Samsung Galaxy S20 is capable of reading significantly lower signal strengths than the remaining devices, thus detecting a larger number of transmitters. In comparison, the cheaper and older smartphone Samsung Galaxy A12 detected significantly less access points within the deployment, partially since it does not support the Wi-Fi standards operating at 5 GHz bands.

### 4.2 Survey methodology

**Fig. 7:**
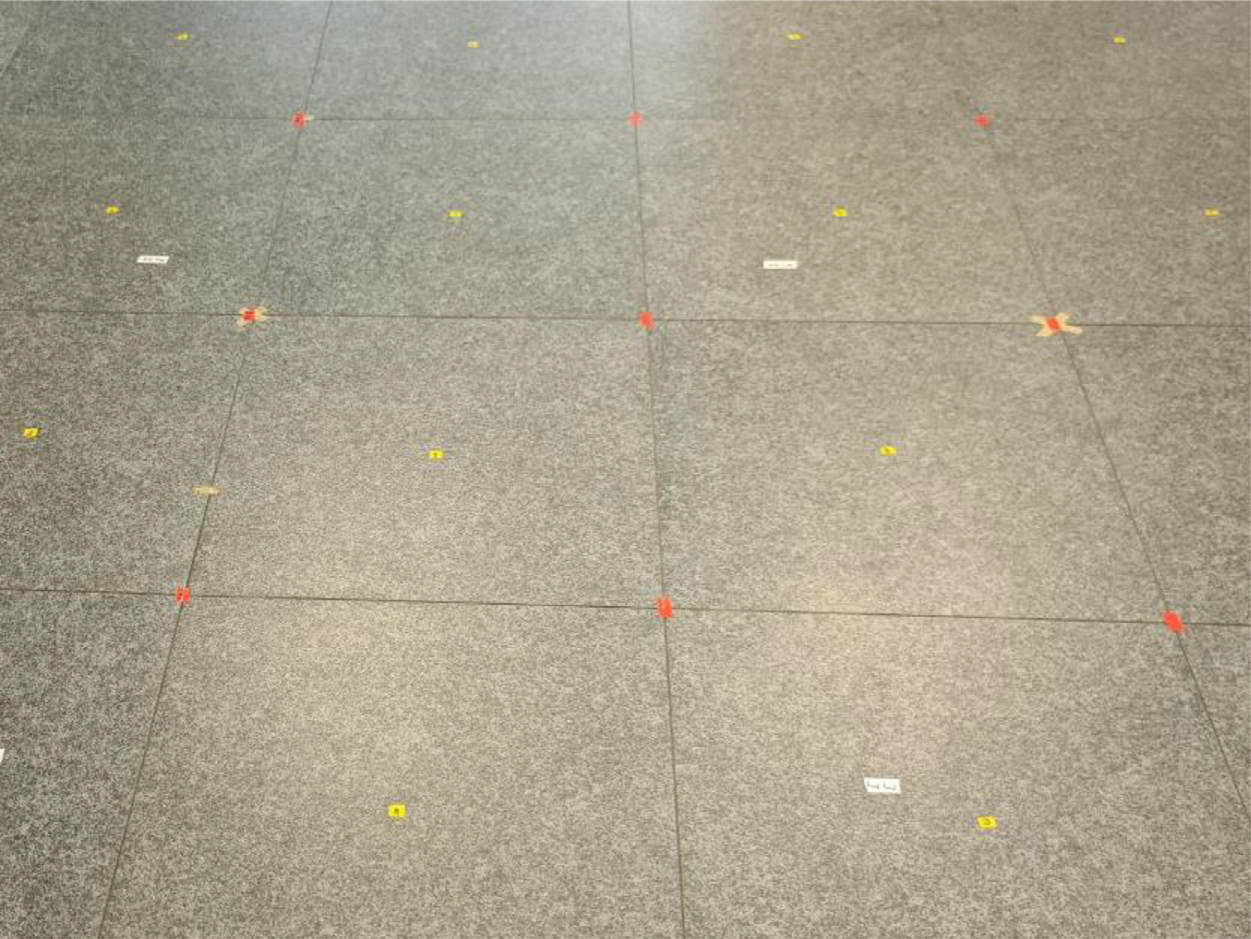
Detail of the two grids. The first, red grid was created at the corners of the rectangular tiles of approx. 60 by 60 cm, while the second grid (yellow) was marked at the center of each tile. Each sticker was marked with the corresponding sequence label.

**Fig. 8:**
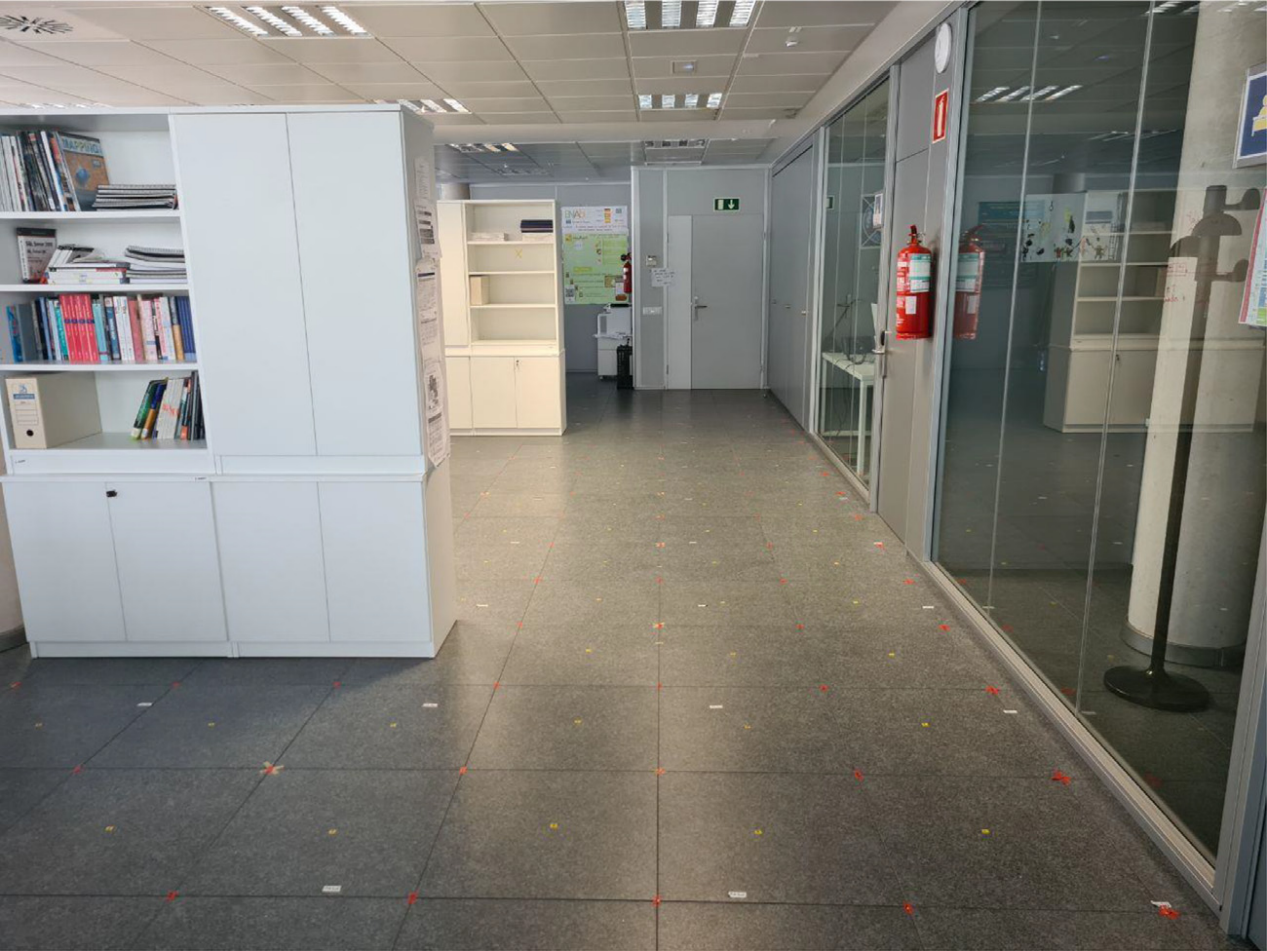
The deployment area, where the measurement took place. The image was taken in the large office on the left side of the deployment, with two small office rooms visible at the right side of the figure.

The samples were measured in two rectangular grids across the deployment, excluding the locations blocked by furniture, walls, and the two inaccessible rooms. The first grid was physically specified by the corners of the regular, rectangular tiles within the whole area, with the dimensions of approx. 60 by 60 cm. The second grid was specified at the centers of the same tiles. Both grids are depicted in Fig. 7, while Figure 8captures part of the environment where the site-survey took place. The accuracy of the coordinates was ensured by carefully measuring the site with a laser measure BOSCH Professional GLM 120 C and systematic markings (as specified in “map2coordinates.xlsx”, sheets “map2coords train” and “map2coord test”) across the whole deployment, enabling iterative surveying using the GetSesorData application.

With this strategy, we created a honeycomb-like structure visible e.g. in Fig. 7 and Fig. 8. The site-survey was performed by the authors of this work to ensure accuracy, in several consecutive days, at times outside of main working hours to limit interference from other devices and ease the surveying process.

**Fig. 9:**
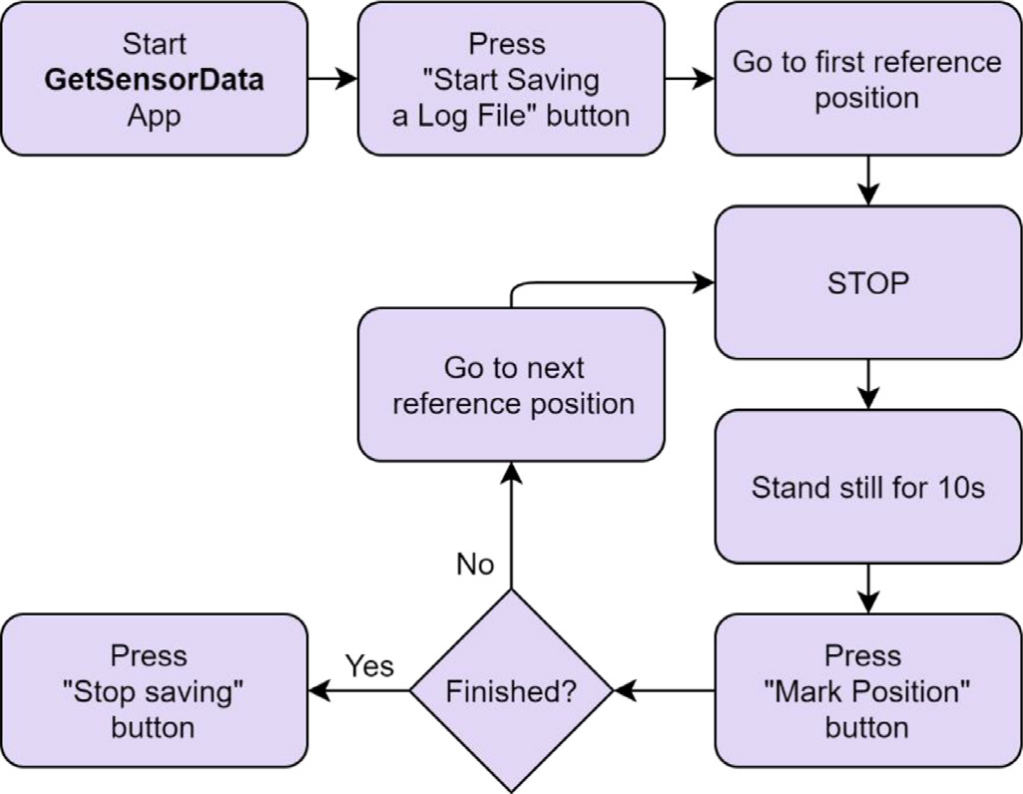
Activity diagram describing the step-by-step data collection process.

The samples were obtained by manually measuring both grids with each device using continuous data logging with the GetSensorData application [1], stopping at every point for at least 10 s before making the measurement tag. The measured device was held in natural pose in front of the surveyor, as if normally using the device. The measurement was marked when the device was situated directly above each location label. The orientation of the user remained systematic throughout the survey process, always forward-facing according to the direction of the sequence specified in “map2coordinates.xlsx” file. The activity diagram providing the step-by-step data collection is presented in Fig. 9. Due to the area of the deployment, each device's measurement campaign was split into a number of individual logs while marking the points they surveyed to ensure consistency. The individual logs are included as the raw data after their anonymization. The pre-formatting algorithm then finds the instance of marking each measured point and finds all Wi-Fi RSSI measurements acquired within 3 s previous to making the corresponding tag in the log. Such data cleaning leave only the last relevant radio measurement array linked to each location tag.

**Fig. 10:**
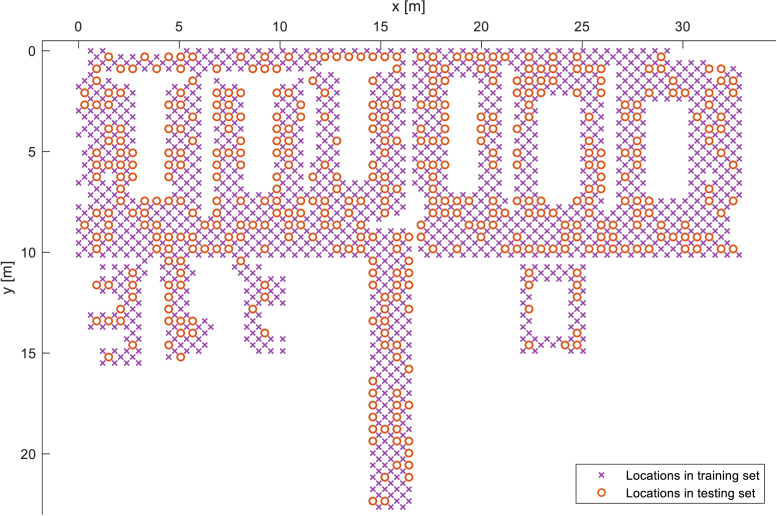
Visualization of samples split between training and testing sets.

In the creation of the formatted Wi-Fi fingerprinting dataset, all samples (fingerprints) corresponding to points in the first grid and samples corresponding to 50% of the points from the second grid (chosen at random) were dedicated to form the training set of features and labels, while the second half of points from the second grid is used as the testing set of data. In other words, the first grid was entirely used for training, while the second grid was randomly split 50:50 between training and testing sets to ensure thorough coverage of the deployment area with training data, while maintaining a random component in the testing set's distribution. Fig. 10 visualizes the split, coherent across all utilized devices, where the samples follow the split with the resulting 76:24 train/test ratio (instead of 75:25, since the number of samples in the second grid is marginally lower). The dataset is presented in a form of MATLAB structure denoted “database”, containing four matrixes, namely “trainingMacs”, “trainingLabels”, “testMacs”, and “testLabels” with the corresponding data. The names were chosen for consistency with previously published indoor positioning databases in [3], where “trainingMacs” and “testMacs” correspond to the training and the testing radio maps, respectively, where each column represents the measurements from a single access point and each row represents the measured array. The missing measurement value is set to 100, while the detected measurements range from −100 to −35 dBm. Marking missing measurement values (i.e., by using 100 in our case) is crucial to clearly distinguish them from values with poor signal quality, as the lack of data denotes an adverse event, such as a received signal strength below the detectable threshold, a temporary outage of the AP, or any other (unknown) event. The training labels (“trainingLabels”) and testing labels (“testLabels”) are arranged so that the columns represent the x-coordinate, y-coordinate, z-coordinate, floor label, building label, and the device label, specified in Table 2. Note that the formatting algorithm merely restructured the data points without altering their intrinsic values, according to the previously mentioned literature for convenience of future use.

### 4.3 Benchmark performance and impact

We include two positioning benchmarks when utilizing the dataset to enable smooth evaluation of the future methods. The TUJI1 dataset was evaluated using the scripts available in [7], while applying the *k* – Nearest Neighbors (*k*NN) classifier onto the data. *k*NN was selected due to its wide usage in the indoor positioning community as a simple yet powerful positioning algorithm. Its main advantage over most machine learning models includes its independence of random initialization, consequently always leading to the same numerical results. Additionally, despite its simplicity it generally provides solid results, often hard to match with other methods. We do not consider other data-driven or machine learning algorithms as benchmarks due to their dependence on the random initialization of the model. Other traditional positioning algorithms, such as trilateration, require known AP coordinates, which are not available within the data. The first benchmark, denoted *1*NN shows the positioning performance when applying a generic model with no parameter tuning, i.e. the benchmark for expected performance of an untuned model. The second benchmark, denoted *Best Coef.*, represents the best positioning performance with respect to the 3D positioning error of the fine-tuned *kNN* algorithm, following the sweep performed in [3], where multiple data representations, distance metrics, and numbers of neighbours were tested to find the best-performing combination of parameters. It determines the fine-tuned model's positioning performance. Table 3 contains the information regarding the parameters and the achieved positioning performance for both benchmarks.

**Table 3:** TUJI1 benchmark performance and settings.

Benchmark	*1*NN	*Best Coef.*
Data representation	Positive	Exponential
Distance metric	Euclidean	Sørensen
Number of neighbors [-]	1	7
Mean 3D positioning error [m]	3.34	2.27

As shown in Table 3, the several-meter positioning accuracy was achieved using both benchmark solutions, yet the *Best Coef.* setting reduced the mean positioning error by 32%, compared to the *1NN* benchmark. The achieved positioning performance reflects the capabilities of utilizing the signals of opportunity, such as Wi-Fi, for localization purposes.

The provided dataset can be freely adjusted to represent the device-specific or cross-device performance by simply filtering the samples using the device label, e.g. study the device-wise generalization properties of the data removing the samples corresponding to the device e.g. “1” from the training data and leaving only the samples corresponding to that device within the testing set.

There are numerous references in the indoor positioning and machine learning domain that could gain immediate benefit from utilizing TUJI1. For example, the dataset perfectly fits the *k*NN positioning and granularity study in [4], as well as the device heterogeneity evaluation utilizing a deep learning model in [9]. Similarly, TUJI1 can be directly applied in a crowdsourcing-based work [10] focusing on quantization and compensation of device heterogeneity effects.

## Limitations

The existence of 310 unique Wi-Fi access points, as well as other users in a limited area likely caused some extent of signal interference, introducing inaccuracies to the network measurements. As the site-survey was performed by hand, slight inaccuracies could have been caused by imperfect positioning of the device above the mark on the ground, despite surveyors’ best efforts. Human body blockage, the device's angle during the measurement, the surveyor's height, and many other real-world aspects could further influence signal variations in measurements. In our future work, additional evaluation of each device's capabilities may show that some phones/tablets struggle more than others to accurately localize the user. Further granularity studies will also be performed to assess the requirements on fine-grained site-surveying in the scope of Wi-Fi-based positioning.

## Ethics Statement

The authors have read and followed the ethical requirements for publication in Data in Brief and confirm that the current work does not involve human subjects, animal experiments, or any data collected from social media platforms.

## CRediT authorship contribution statement

**Lucie Klus:** Conceptualization, Methodology, Software, Data curation, Writing – original draft, Visualization. **Roman Klus:** Conceptualization, Methodology, Software, Data curation, Writing – original draft. **Elena Simona Lohan:** Resources, Writing – review & editing, Supervision, Project administration, Funding acquisition. **Jari Nurmi:** Supervision, Project administration, Funding acquisition. **Carlos Granell:** Resources, Supervision. **Mikko Valkama:** Writing – review & editing, Supervision, Project administration, Funding acquisition. **Jukka Talvitie:** Resources, Writing – review & editing, Project administration, Funding acquisition. **Sven Casteleyn:** Resources, Writing – review & editing, Funding acquisition. **Joaquín Torres-Sospedra:** Conceptualization, Methodology, Software, Formal analysis, Writing – review & editing, Funding acquisition.

## Data Availability

TUJI 1 (Original data) (Zenodo).
